# Computationally Efficient Automatic Coast Mode Target Tracking Based on Occlusion Awareness in Infrared Images

**DOI:** 10.3390/s18040996

**Published:** 2018-03-27

**Authors:** Sohyun Kim, Gwang-Il Jang, Sungho Kim, Junmo Kim

**Affiliations:** 1 Agency for Defense Development, P.O. Box 35, Yuseong, Daejeon 34186, Korea; gijang@add.re.kr; 2 Department of Electronic Engineering, Yeungnam University, 280 Daehak-ro, Gyeongsan, Gyeongbuk 38541, Korea; sunghokim@ynu.ac.kr; 3 Department of Electrical Engineering, KAIST, 291 Daehak-ro, Yuseong-gu, Daejeon 34141, Korea; junmo@ee.kaist.ac.kr

**Keywords:** infrared image, automatic tracking, electro-optical tracking system, coast mode tracking

## Abstract

This paper proposes the automatic coast mode tracking of centroid trackers for infrared images to overcome the target occlusion status. The centroid tracking method, using only the brightness information of an image, is still widely used in infrared imaging tracking systems because it is difficult to extract meaningful features from infrared images. However, centroid trackers are likely to lose the track because they are highly vulnerable to screened status by the clutter or background. Coast mode, one of the tracking modes, maintains the servo slew rate with the tracking rate right before the loss of track. The proposed automatic coast mode tracking method makes decisions regarding entering coast mode by the prediction of target occlusion and tries to re-lock the target and resume the tracking after blind time. This algorithm comprises three steps. The first step is the prediction process of the occlusion by checking both matters which have target-likelihood brightness and which may screen the target despite different brightness. The second step is the process making inertial tracking commands to the servo. The last step is the process of re-locking a target based on the target modeling of histogram ratio. The effectiveness of the proposed algorithm is addressed by presenting experimental results based on computer simulation with various test imagery sequences compared to published tracking algorithms. The proposed algorithm is tested under a real environment with a naval electro-optical tracking system (EOTS) and airborne EO/IR system.

## 1. Introduction

Automatic tracking of an electro-optical system refers to keeping a target at the center of the images by driving the sensor’s LOS (line of sight) according to the result of a video tracker that calculates a change in the target’s position using acquired images. As shown in Figure 1, a video tracker detects a target’s location from input images and sends the pixel error (difference between target’s location and center in the image) to the servo controller. Then, the servo controller drives gimbals as a mount of tracking error to locate the target at the center of images. Iteration of that process keeps a target in the sensor’s line of sight. These kinds of automatic tracking systems have a variety of uses in military and security systems because they provide automotive operation and a target’s accurate position simultaneously.

Although image tracking has been the focus of many studies over the world, there are still many challenging issues such as overcoming target’s occlusions, appearance changes, significant motions, background clutters, etc. Among the challenges in the image tracking area, the occlusion problem is one of the most crucial problems.

In this paper, a novel automatic coast mode tracking method is proposed to prevent from target loss against the target occlusion, which is one of the functions for improving the operability of centroid tracker for infrared images, considering the computational cost of fast moving target tracking. The proposed algorithm consists of three steps. The first step is to make a decision to enter the coast mode that estimates when the target occlusion occurs. The second step is to perform memory tracking using a prediction result of the target’s location rather than image tracking result during the target’s blind time. The last step is to re-lock the target (or re-acquisition target) after the target occlusion is released. This study shows effective performance according to the results based on computer simulations with various test imagery compared with several published tracking algorithms. It is tested under a real environment with an electro-optical tracking system (EOTS) mounted on ship and airborne EO/IR (electro-optic/infrared) system.

Section 2 introduces the background of coast mode tracking and the proposed occlusion aware–coast mode tracking (OA-CMT) method is explained in Section 3. In Section 4, experimental results through the simulation and real EOTS dataset validate the feasibility of the proposed method in various occlusion scenarios. Section 5 concludes this paper.

## 2. Background of Coast Mode Tracking

A video tracker finds a target’s location in the acquired images from imaging sensors and calculates tracking error using image center information. There is a problem of tracking loss affected by abrupt obstacles, because the tracking result is computed depending on the brightness of each pixels of image. There have been various attempts to solve the occlusion problem in tracking. For example, previous methods using appearance models [1,2,3], adaptive appearance modeling by statistical analysis [4,5,6], and template matching of target models [7,8] showed good performance for limited situations, but it is easy to degrade tracking performance because blind updates in long-term occlusion status causes contamination of appearance models. Multi-camera-based methods [9,10,11] were also proposed, but these methods require a complex multi-camera setup with high cost, which is not appropriate for a moving camera platform such as EOTS. Patch matching-based methods [12,13,14] can widely handle not only the occlusion problem but can also overcome appearance changes. However, they can deal with a short-duration of partial occlusion and fail at severe occlusions lasting for a long time. Probabilistic approaches [15,16,17,18] and L1 trackers [19,20] have been proposed several times. State-of-the-art trackers, upgraded correlation trackers [21] and neural network-based tracking methods [22,23,24], have been presented, which show outstanding tracking performance in multiple target tracking, cluttered environments, changing of target’s shape, and occlusion. However, these trackers require an on-line or off-line training process with an amount of data sets of targets to accommodate various situations, meaning they are not suitable for tracking systems that must track arbitrary targets what the operator chooses.

The aforementioned techniques have two limitations in EOTS applications. One is the computational cost for real time processing on embedded system. They need more than 30 frames per second to track a fast-moving target. Algorithms using complex calculation are useless for on-line systems. The other problem is that those kinds of tracking method are not applicable very well in the case of infrared images because they deal with features of imagery with clear edge or color information. Infrared sensors, recording radiant intensity that is emitted by a targets’ thermal energy, are superior to visible cameras in detecting and tracking small objects at long distance and can be used at night time, which leads to the wide use of infrared sensors in military applications. However, it is difficult to extract the feature points for tracking because the shapes of targets are usually shown blurred and blinked due to atmospheric scattering and air turbulence between the target and sensor. Therefore, it is better to use target intensity information for target detection and tracking in infrared images. The targets to track are normally mapped to high intensity regions compared to the surrounding background.

The proposed algorithm can overcome the target occlusion status using centroid trackers for infrared images where the extraction of meaningful feature is difficult. The centroid tracking method, using only the brightness information of an image, is still used widely in infrared tracking systems because of its computational simplicity.

**Figure 2:**
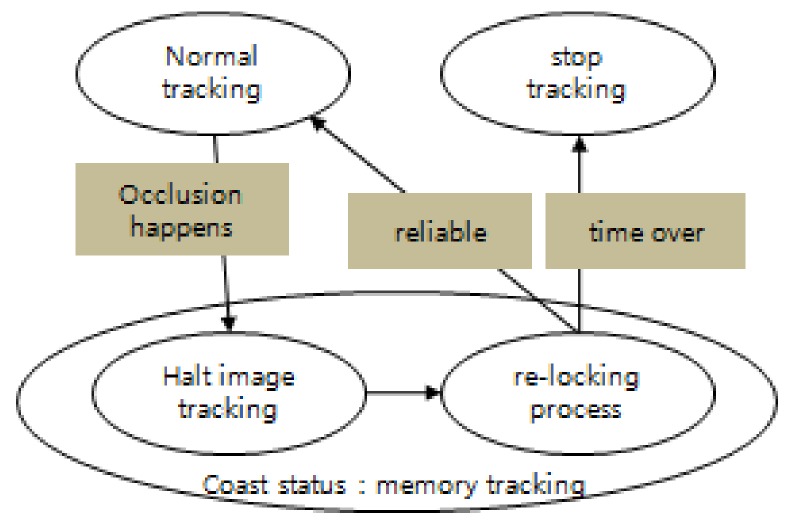
State diagram of the coast mode tracking process.

The coast mode refers to one of the tracking modes preventing tracking loss against a target’s occlusion [25]. As shown in Figure 2, the image tracker is halted for a while and turned to a memory tracking status when the reliability of tracking becomes lower or the prediction of target occlusion event happens on normal tracking status. The memory tracking refers to the status that a servo drives a sensor’s line of sight in the predicted direction and speed from movement data is measured in normal tracking status before the occlusion. Using predicted results rather than image tracking results during occlusion status, which obtain meaningless results from the image tracker, helps to increase of the possibility of resuming tracking after long-term occlusion while some trackers suffer from maintaining image tracking over long periods of occlusion. After the blind time, it tries to find the target to resume video tracking. When the target can be detected and becomes reliable enough to be re-locked, image tracking is started. Otherwise, the video tracker stops if it fails to re-lock on the target after a certain period of time.

In general, the coast tracking mode could be divided into forced coast tracking, automatic coast tracking [25], and gun coast tracking [26] according to the system requirement. Forced coast tracking runs by user’s decision and operation; it is also referred to as manual coast tracking mode. When the operator selects forced coast mode, it starts memory tracking without image tracker until he locks on the target to track. The automatic coast tracking means the automatic process of coast tracking for user’s convenience, so the decision algorithm is required to enter coast status and use the re-locking algorithm with image processing. The gun coast tracking is used to avoid tracking loss caused by gun firing. It enters coast status automatically with gun fire command input. In this case, the target re-locking process is tried after the fixed time that is expected for disappearing gun smoke.

## 3. Proposed OA-CMT Based Infrared Target Tracker

**Figure 3:**
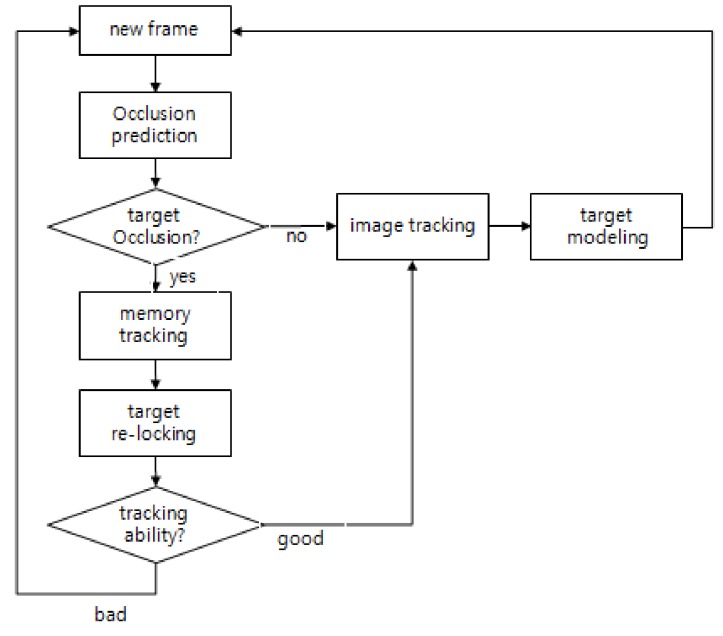
Flowchart of the coast mode tracking.

The proposed occlusion-aware coast mode tracking (OA-CMT) is performed in the order shown in Figure 3. In a newly inputted frame image, it predicts if the target is occluded by an obstacle through image processing around the target being tracked. When there is no occlusion, it continues image tracking and collects information of the brightness, shape and motion of the target. If any obstacle is detected around the target, it stops image tracking, switches to memory tracking status, predicting the target’s location using historically filtered data, and then tries to reacquire the target. When the occlusion is ended and the target candidate is detected, the image tracking is resumed if the image tracking result is reliable. Otherwise, it will continue maintaining memory tracking and attempting to reacquire the target.

### 3.1. Prediction of Target’s Obstruction

The awareness of target occlusion is made by two steps. In the first step, the “target likelihood obstacle check” predicts a target’s occlusion by checking the brightness similarity of the background in the direction of the target’s movement: if the background brightness around a target is similar to that of the target’s, the centroid tracker would miss the target. In the second step, “background screening check” predicts the loss of the tracking situation where the background appears which can hide the target. If any target likelihood obstacles and background likelihood obstacles are detected, it stops the centroid tracking for the target, turns the tracking status to the coast mode and starts memory tracking.

**Figure 4:**
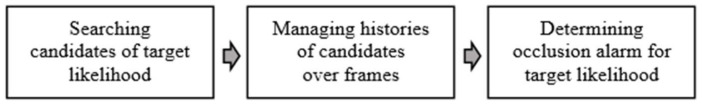
Procedure of target likelihood brightness check.

The target likelihood obstacle check consists of the steps of searching for targeted candidates with similar brightness, carrying the history of the candidates over frames, and determining the occlusion alarm for target likelihood, as shown in Figure 4.

**Figure 5:**
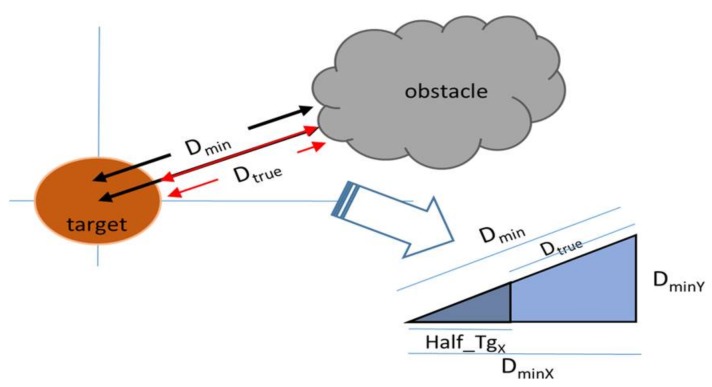
Calculating true distance (D_true_) between the target and an obstacle.

During the tracking, it is more likely to be affected by an object which has similar brightness to the target and is located close to the target. The first step, searching for candidates, is to detect the objects of particular brightness that are likely to be classified as a target by the centroid tracker. For the efficiency of computation time, it is necessary to set an appropriate searching area around the target and divide it into several blocks. The brightness comparison for screening the target likelihood area is performed on a block related to the target size (set as a quarter of the target’s size) rather than on a pixel-by-pixel basis. The searching area is set to a multiple of the block’s size considering target’s speed and moving direction. Then, it classifies an area as a “bright block” if the count of pixels brighter than the threshold value is more than 50% of the block area. Then, it registers a candidate by clustering the adjacent “bright blocks” with the information such as the size, location, average brightness, and the “true distance (D_true_)”. True distance means the distance between the outer surfaces of target and obstacle, as shown in Figure 5. With the similar triangles rule, D_min_ (minimum distance from center of the target to outer surface of obstacle) and (D_min_ − D_true_) have a proportional relation with their x-components, D_min_ and Half_TgX_ (half of target size), then D_true_ could be approximately calculated as Equation (1).

(1) $$D_{\mathrm{true}}=D_{\min}\times \frac{\left(D_{\mathrm{minX}}-{\mathrm{Half}}_{\mathrm{TgX}}\right)}{D_{\mathrm{minX}}},$$

The second step is to manage the histories of each candidate over frames to predict whether candidates affect the centroid tracking process. The matching of candidates between the current frame and previous frame is performed by comparing the registered information with the effective distance. In this step, the process is carried out with three cases: (1) the observed candidate having no history (that means it is first observed candidate); (2) having history but not being an observed candidate (that means a disappeared candidate); (3) observed candidate having history. If a candidate has history, the effective distance that the candidate is expected to move in the next frame is set to manage the change trend of the candidate over frames and used for matching between the observed candidate and history in the next frame.

The occlusion alarm is determined by the candidate’s approaching distance to the target. First, it computes average velocity for each of the candidates, calculates the “estimated distance (D_est_)” that reflects the prediction of the obstacle’s movement on the D_true_, such as in Equation (2), then checks if the estimated distance is smaller than a distance threshold value. At this time, the distance threshold value is set to be proportional to the block size considering the block unit operation in the first step. It makes an alarm of target likelihood occlusion when the candidate is present and the estimated distance is smaller than the threshold value.

(2) $$D_{\mathrm{est}}=D_{\mathrm{true}}+\mathrm{ScaleFactor}\times \left(D_{\mathrm{true}}-D_{\mathrm{true},\;\mathrm{history}}\right),$$

**Figure 6:**
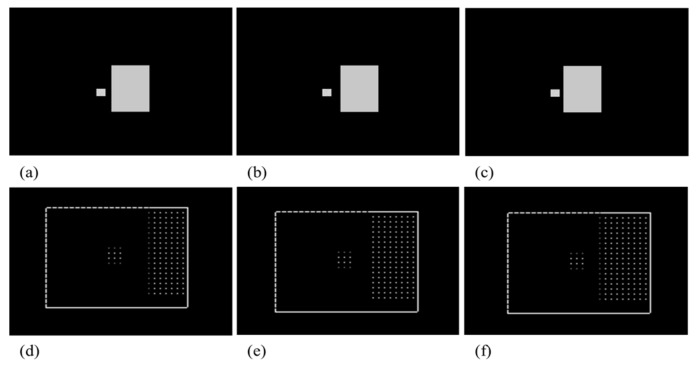
Result of target likelihood obstacle check in test image sequence (**a**–**c**) are test image sequences, and (**d**–**f**) show the results of searching candidates for (**a**–**c**) with magnifying interested area. In (**d**–**f)**, the square box represents the search area spots, meaning a bright block. Calculation result of estimated distance is (**d**) 16, (**e**) 11, (**f**) 6.

Figure 6 shows the results of the target likelihood obstacle check in the test image sequence which is made to for computer simulation. (a–c) are partial views of a test image sequence where a 16 × 16 pixel-sized target approaches the obstacle with an average brightness of target. (d–f) are the results of searching candidates for (a–c) with magnifying interested area. In (d–f), the square box represents the search area, and a white spot means a bright block. The operational interval of blocks could be guessed from the distance between spots. There are two clusters of spots: the smaller one is the target and the bigger one is the obstacle. The calculation results of estimated distance are 16, 11, 6 for (d–f), respectively. An occlusion alarm is made at (f).

**Figure 7:**
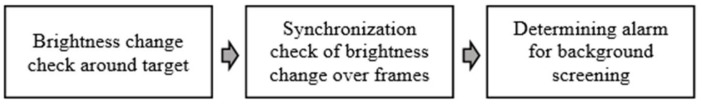
Procedure of background screening check.

The background screening check is a process to determine if the track is difficult to keep because an obstacle located between the IR camera and the target obscures the view of the camera even though its brightness is not similar the target. It consists of three steps: the first step is the brightness change check of four guard gates and four background gates as shown in Figure 7. The second step is to make a pre-alarm with a synchronization check of sequential occurrence of brightness changes between guard gate and background gate. The last step is to determine the occlusion alarm for background screening with pre-alarm and size tracking window. Figure 7 presents the procedure of the background screening check.

**Figure 8:**
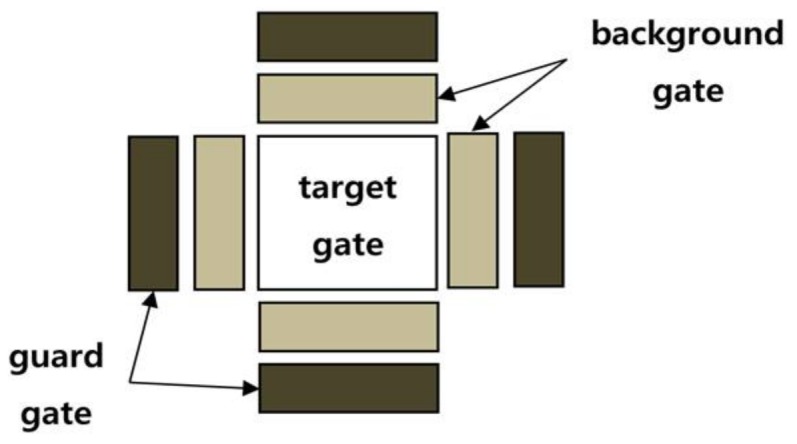
Shape of background gates and guard gates. There are four gates for each direction: up, down, left, and right side around target. Both background gate and guard gate are 1/4 size of target gate.

**Figure 9:**
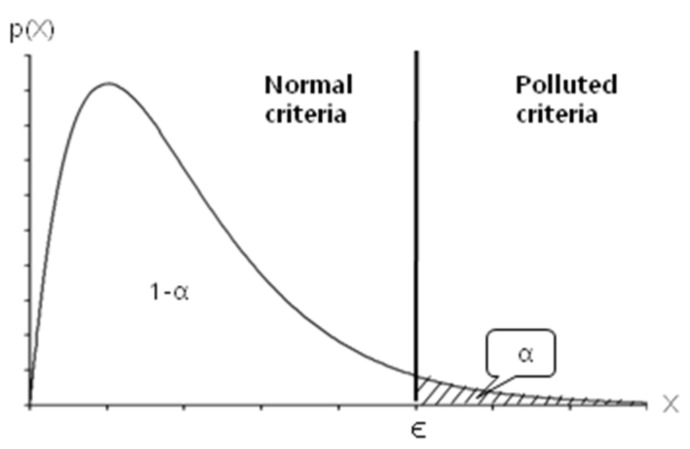
Cumulative distribution function of χ^2^ distribution.

During the first step, brightness change around the target is determined using χ^2^ distribution for their brightness. First of all, four background gates and four guard gates about four directions are set around the target as shown in Figure 8. The cumulative distribution function (CDF) of average (μ) and standard deviation (σ) for brightness on each gate over frames are computed. As shown in Figure 9, a region of α sized area is set as polluted criteria that brightness change is detected; otherwise, the region of (1–α) sized area is set normal criteria. As represented in Equation (3), if the chi-square distribution value is smaller than ϵ, it is determined as a normal situation, otherwise the gate is determined to be polluted by an obstacle in the case of greater than ϵ. To solve the scintillation problem of IR imagery, applying a normalization factor considering global intensity is recommended, as in Equation (3).

(3) $$p\left(X\in \mathrm{normal}\;\mathrm{status}\right)=p\left(\frac{{\left[X/\mathrm{NF}-\mu \right]}^{T}}{\sigma }\sum^{-1}\frac{\left[X/\mathrm{NF}-\mu \right]}{\sigma }\leq \epsilon \right)\;p\left(X\in \mathrm{pollusion}\;\mathrm{status}\right)=p(\frac{{\left[X/\mathrm{NF}-\mu \right]}^{T}}{\sigma }\sum^{-1}\frac{\left[X/\mathrm{NF}-\mu \right]}{\sigma }\geq \epsilon ),$$

In the second step, a pre-alarm is made when the synchronicity of polluted gates with the sequence is admitted. There is an observed sequence because the first guard gate is polluted and then the background gate in the same direction will be polluted within several frames when an obstacle approaches to the target. Synchronization of gate pollution is determined more accurately when considering a target’s moving direction and speed.

The last step is to make a final decision of the background screening check regarding the size reduction of the target gate of the same direction with gate pollution under pre-alarm situation.

**Figure 10:**
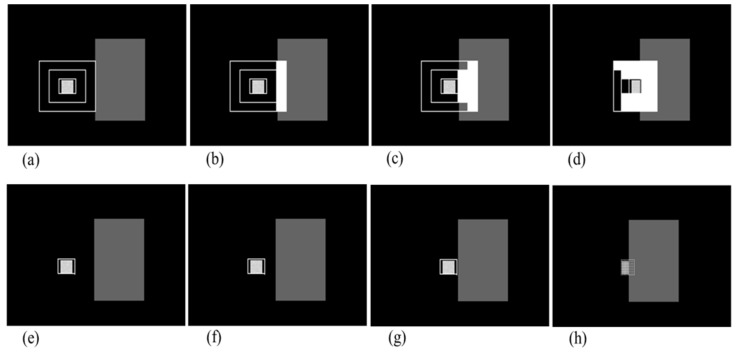
Result of background screening obstacle check in test image sequence. (**a**–**d**) show the process of gates’ pollution over frames by obstacle; (**e**–**h**) present the status of image tracking. In (**h**), the background screening alarm finally occurs.

**Figure 11:**
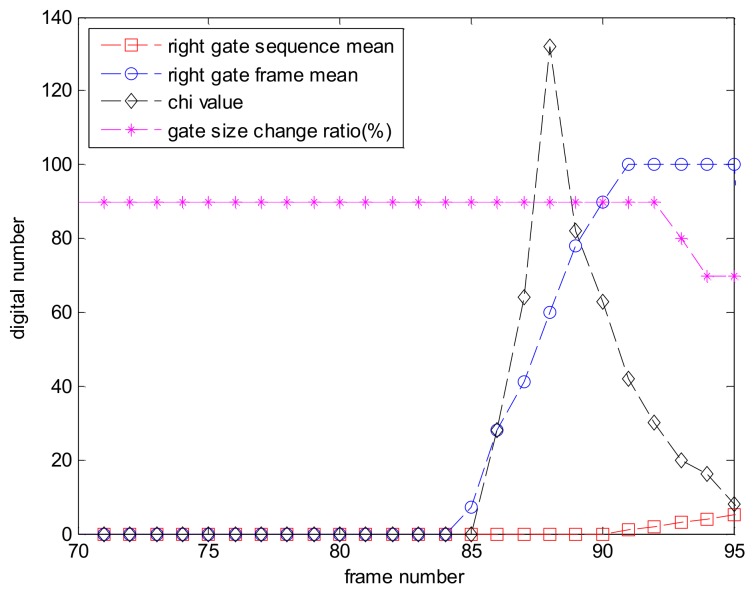
Brightness change histories of the right side gate among four-directional background gates are shown when the target moves in the right direction. χ value (chi) increases as the intensity mean of the frame mean (right gate frame mean) definitely changes compared to intensity mean over the sequence (right gate sequence mean). As the target is being hidden behind an obstacle, gate size became smaller than its average size.

Figure 10 shows the result of the background screening check for a test image sequence which is made to perform a computer simulation, as in the case of a target likelihood obstacle check but with a darker obstacle than target. (a–d) show the process of gates’ pollution over frames by obstacle, while (e–h) present the status of image tracking. With this sequence, the brightness of the right side of the target changes as the target approaches the obstacle, as in Figure 11. χ value increases when the intensity mean of the frame (*x*-axis) of the background gate definitely changes compared to intensity mean over sequence (μ). As the target is being hidden behind the obstacle, the gate size becomes smaller than its average size. With those clues, it can be seen that background screening alarm occurs in Figure 10h, as the target tracking gate disappears because the obscuring alarm makes image tracking stop.

### 3.2. Memory Tracking Using Tracking Filter

If a target’s obstruction is predicted with the process described in the previous chapter, it stops the image tracker and performs memory tracking with inertia until the target is re-locked. In the memory tracking status, the image tracker cannot output the pixel error, and the target’s location must be predicted with tracking filter. Of course, it is possible to acquire more accurate location of the target using advanced tracking filters such as the interactive multiple model (IMM) filter [27] or particle filter with three-dimensional information [28] if it can obtain the distance to the target. However in this study, α-β-γ filter [29] is simply selected to get a position for re-locking process without range information.

The α-β-γ filter is a simplified form of the Kalman filter for estimation, data smoothing and control applications. It is suitable for predicting a target’s location in memory tracking because it keeps the effect of Kalman filter in a time-invariant state without a detailed system model. Starting from a one-dimensional, linear, time-invariant, ideal target motion model described in Equation (4), where y(k) is the target state vector (composed of location x(k), velocity v(k), and acceleration a(k)) at time k, w(k) is the unknown target maneuver, and their transition matrix φ(k), ψ(k). Normally, measurement z(k) is composed by sum of position and noise n(k) as in Equation (5), the target prediction and correction are solved like Equation (6), and prediction Equation (7).

(4) $$y\left(k+1\right)=\varphi \cdot y\left(k\right)+\psi \cdot w\left(k\right)\;\left[\begin{array}{c} x\left(k+1\right)\\ v\left(k+1\right)\\ a\left(k+1\right) \end{array}\right]=\left[\begin{array}{ccc} 1 & T & T^{2}/2\\ 0 & 1 & T\\ 0 & 0 & 1 \end{array}\right]\left[\begin{array}{c} x\left(k\right)\\ v\left(k\right)\\ a\left(k\right) \end{array}\right]+\left[\begin{array}{c} T^{2}/2\\ T\\ 1 \end{array}\right]w\left(k\right),$$

(5) $$z\left(k\right)=h\cdot y\left(k\right)+n\left(k\right)\;z\left(k\right)=\left[\begin{array}{ccc} 1 & 0 & 0 \end{array}\right]\left[\begin{array}{c} x\left(k\right)\\ v\left(k\right)\\ a\left(k\right) \end{array}\right]+n\left(k\right),$$

(6) $$\overset{ˇ}{y}\left(k+1|k+1\right)=\overset{ˇ}{y}\left(k+1|k\right)+K\left(k+1\right)[z\left(k+1\right)-h\cdot \overset{ˇ}{y}\left(k+1|k\right),$$

(7) $$\overset{ˇ}{y}\left(k+1|k\right)=\varphi \cdot \overset{ˇ}{y}\left(k|k\right),$$

(8) $$K=\left[\begin{array}{c} \mathrm{Kx}\\ \mathrm{Kv}\\ \mathrm{Ka} \end{array}\right]=\left[\begin{array}{c} \alpha \\ \beta /T\\ \gamma /T^{2} \end{array}\right]$$

K, steady state Kalman gain, is related to the α-β-γ parameters as shown in Equation (8). The optimal α-β-γ relationships are previously reported [29].

**Figure 12:**
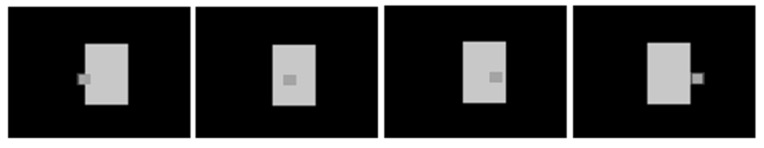
Result of memory tracking using α-β-γ filter in test imagery sequences. Dotted square is the prediction of target’s position.

A tracking filter is implemented using the tracking index parameter of time period T = 1/30 s, target maneuverability = 3 g (m/s^2^), and measurement noise = 0.2 m. The tracking result with test imagery sequences is shown in Figure 12. While the target is under occlusion, the location of the target is predicted by the α-β-γ filter. The dotted square represents the prediction of the target’s position regarded as being hidden by an obstacle in Figure 12. The performance of the filter can be verified from the result that the dotted square appears superimposed on the location of the target after blind time.

### 3.3. Target’s Re-Locking

When the target is seen in the image after blind time, the process of re-locking target is performed in order to resume image tracking. The proposed method is designed using the target brightness value, which is the main characteristic of infrared images.

Spatial or temporal filters are often applied for target detection in the case of dealing with intensity information. However, these methods are likely to fail to detect targets when the brightness of the target is changed by the AGC (automatic gain control) function of infrared cameras. Therefore, in the proposed study, the computing histogram ratio is used between the target and background.

**Figure 13:**
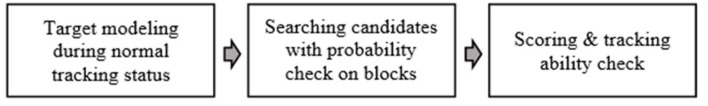
Procedure of target re-locking.

Figure 13 shows the procedure of the proposed target re-locking algorithm. First, the target model is established by gathering information of the target during image tracking. When a decision of occlusion is made, it starts screening the blocks which have a high probability of being the target within the search area divided into blocks. The probability of each block is calculated by the sum of the histogram ratio modeled in the previous step, and blocks having a probability higher than the threshold value are selected as the target candidates. Next, the selected candidates are scored by comparison to target model, and the highest scored candidate is picked as the target.

In the first step of the target re-locking algorithm, on-line training of the target model is performed during image tracking status. This method calculates the histogram ratio between the target gate and four background gates (except guard gates in Figure 8), while the original histogram back-projection method [30] is computed for the entire image. It causes not only heavy computation but also less accuracy because the background histogram of the whole image may change according to the target’s movement.

Equation (9) [31] shows the formula for target model with a histogram ratio of target model H_r_(i), target’s histogram value H_t_(i), sum of background gates’ histogram value, for gray level i. In Equation (9), a high histogram ratio of grey level i means a high probability that level I belongs to the target [32]. To get a more accurate target model, H_r_(i) must be averaged over frames, especially in the case of using a histogram stretched image.

(9) $$H_{r}\left(i\right)=\frac{H_{t}\left(i\right)}{H_{t}\left(i\right)+H_{t}\left(i\right)+1},$$

Other information such as target size, location, shape, and intensity variance are collected and statistically modeled in this step to be applied for the scoring process.

A transition to the coast mode occurs by determining occlusion, and the image process for detecting target candidates is activated until image tracking resumes. The searching candidate process works within the searching area considering target’s moving speed and direction. For reduced computation time and de-noising effect, it is performed by the unit of blocks referred in the previous section, “prediction of target’s obstruction”.

**Figure 14:**
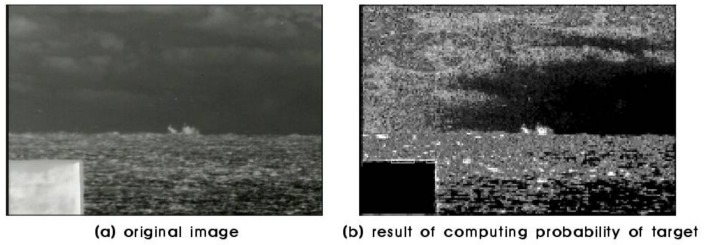
Result of screening target candidates.

For each block, the average is computed of the extracted value of the histogram ratio model corresponding to the brightness of each pixel within the block. This average value presents the probability of how similar the block is to the target. Figure 14b shows the target probabilities of the test image, a higher intensity block presents a higher probability of being the target.

After the probability values are acquired about all blocks, clustering is performed for the adjacent blocks which are screened by the probability threshold. These clusters are regarded as target candidates: bigger or smaller clusters are excluded compared to the target’s size.

In the final step of the target re-locking algorithm, the score is calculated to determine the target to track and decide whether to resume tracking. For the target candidates, the similarity with the target is scored by comparison to the target model including size, intensity variation, aspect ratio, and target probability (i.e., average value of histogram ratio over pixels) that are statically collected in image tracking status. The candidate with the highest score is selected as the target, and the tracking reliability is checked to make a decision whether to switching to image tracking status.

**Figure 15:**
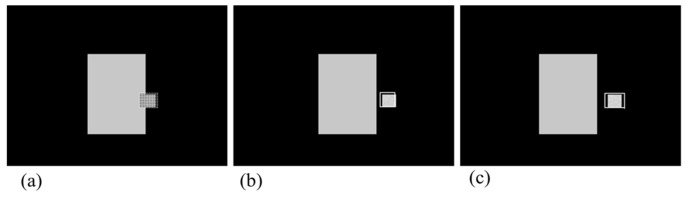
Result of target re-locking. During blind time, the re-locking algorithm is being executed as shown in (**a**). It is shown that the target is re-locked after blind time in (**b**). In (**c**), image tracking is resumed through checking ability of tracking.

Figure 15 shows the result of the target’s re-locking process. Figure 15a shows that the re-locking algorithm is being executed during blind time. After blind time, the target appears, the target gate is located on the target as a result of the re-locking algorithm. Figure 15c shows that image tracking is resumed through the checking track ability.

## 4. Experimental Results

In this section, to verify the performance of the proposed method, OA-CMT, computer simulations have been carried out on several infrared and visible images. Table 1 shows the list of the test imagery sequences including descriptions about sensor types, targets, and obstacles.

**Figure 16:**
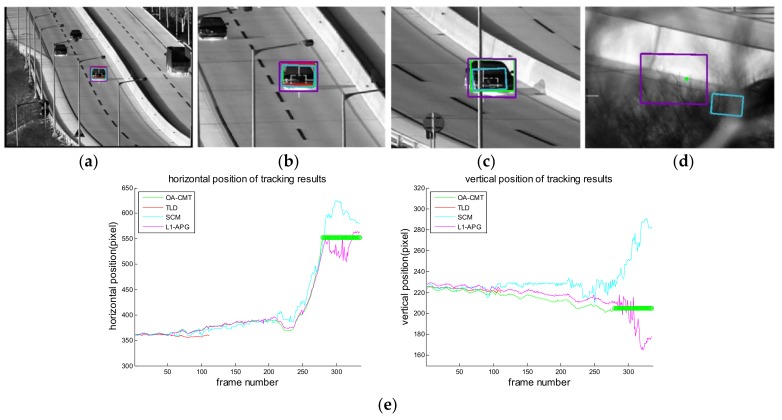
Computer simulation result of imagery ID 1. (**a**) Normal tracking status (original image); (**b**) magnifying interested area; (**c**) TLD loses track while the car is partially hidden by a street lamp; (**d**) OA-CMT turns to memory tracking status because the car is hidden by a bush; (**e**) tracking trajectories of each tracker. The green dot says OA-CMT turns to memory tracking status while other trackers are tracking the wrong position.

**Figure 17:**
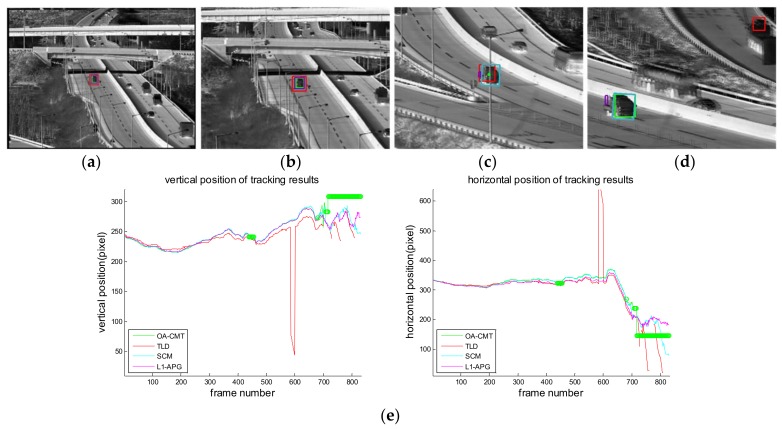
Computer simulation result of imagery ID 2. (**a**) Normal tracking status (original image); (**b**) magnifying interested area; (**c**) L1-APG loses track while the car is partially hidden by a street lamp while OA-CMT turns to memory tracking status and will re-lock successfully after several frames; (**d**) TLD tracks wrong target;(**e**) tracking trajectories of each tracker. Around the 600th frame, TLD presents a very different position because of tracking the wrong target (at the time of (**d**)). After the 700th frame, the car is hidden by trees, OA-CMT turns to memory tracking status, while other trackers give wandering positions.

**Figure 18:**
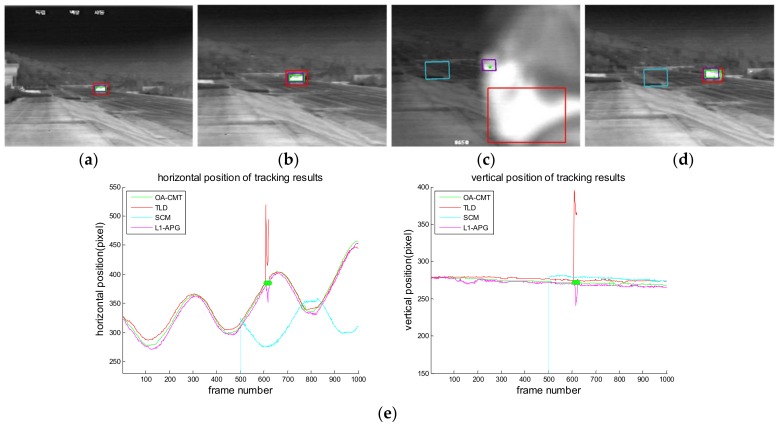
Computer simulation result of imagery ID 3. (**a**) Normal tracking status (original image); (**b**) magnifying interested area; (**c**) OA-CMT turns to memory tracking status when the tank is hidden by a human; (**d**) TLD, L1-APG and OA-CMT re-lock target successfully; (**e**) tracking trajectories of each trackers During blind time, tracking results of TLD are hopping because it tracks the human. SCM lost tracking with the changing shape of the target when tank turned its direction.

**Figure 19:**
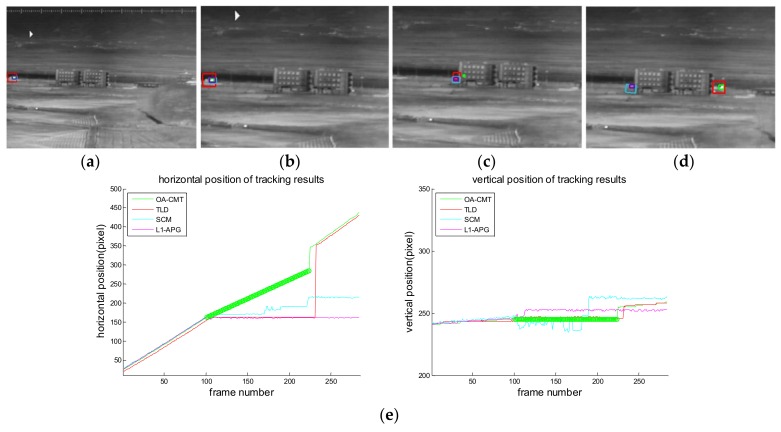
Computer simulation result of imagery ID 4. (**a**) Normal tracking status (original image); (**b**) magnifying interested area; (**c**) OA-CMT turns to memory tracking status as the car is hidden by a building (**d**) TLD and OA-CMT re-lock target successfully; (**e**) tracking trajectories of each tracker. Predicted position of target of OA-CMT has no big difference with the position of the target after blind time.

**Figure 20:**
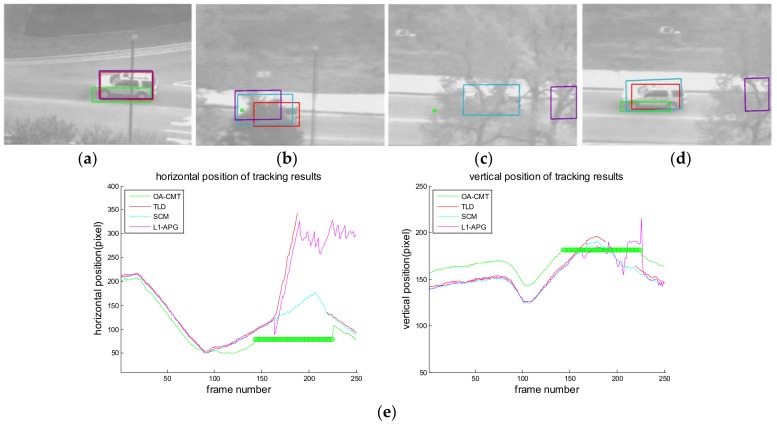
Computer simulation result of imagery ID 5. (**a**) Normal tracking status (original image); (**b**) OA-CMT turns to memory tracking status when the tank is hidden by trees; (**c**) other trackers are wandering; (**d**) TLD, SCM and OA-CMT re-lock target successfully after blind time; (**e**) tracking trajectories of each tracker. TLD follows the wrong target during blind time.

**Figure 21:**
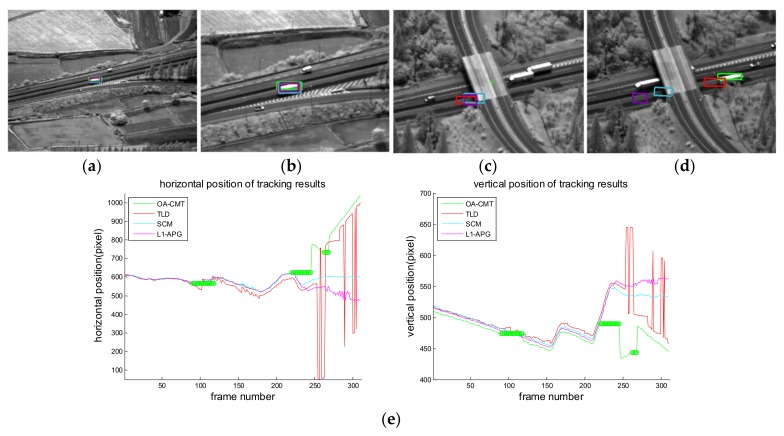
Computer simulation result of imagery ID 6. (**a**) normal tracking status (original image); (**b**) magnifying interested area; (**c**) OA-CMT turns to memory tracking status while the bus is hidden by the overpass (bridge) but other trackers are tracking the bridge; (**d**) OA-CMT re-locks target successfully, TLD re-locks the back side of the target while other tracker loses track; (**e**) tracking trajectories of each tracker. After blind time (from 250th frame) tracking positions of TLD are hopping because it confused the target with the other bus.

**Figure 22:**
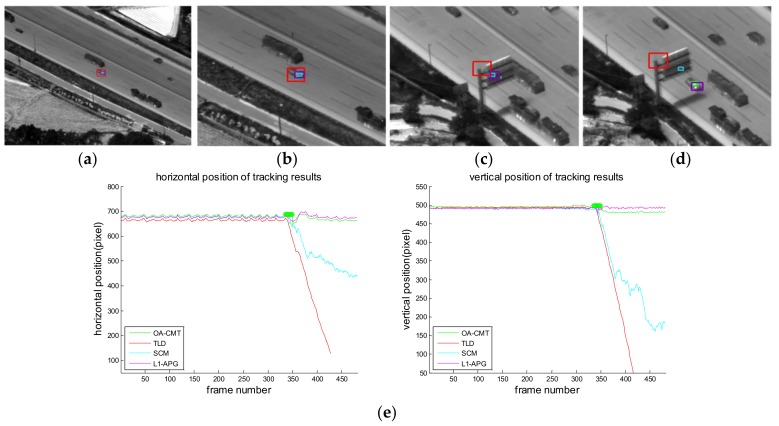
Computer simulation result of imagery ID 7. (**a**) Normal tracking status (original image); (**b**) magnifying interested area; (**c**) OA-CMT turns to memory tracking status as the car is hidden by the traffic sign; (**d**) L1-APG and OA-CMT re-locks target successfully while TLD and SCM lost the target; (**e**) tracking trajectories of each tracker.

Figure 16, Figure 17, Figure 18, Figure 19, Figure 20, Figure 21 and Figure 22 show the results of the computer simulations compared to other published trackers dealing with target occlusion. In each figure, (a–d) are selected scenes showing meaningful results among sequences, including tracking result of Tracking Learning Detection (TLD) [33] (red box), L1 tracker using Accelerated Proximal Gradient (L1-APG) [20] (violet box), Sparsity-Based Collaborative Model (SCM) [18] (cyan box), and OA-CMT (green box and green dot during coast status).

Each tracking result also includes tracking trajectories of vertical and horizontal locations over frames. In the case of OA-CMT, there are no big differences between the predicted results (presented as green dots) and re-locked locations during target occlusion. It is not desirable for EOTS that the tracking result includes abrupt changes of target’s location as in Figure 18, Figure 19 and Figure 22, because the servo controller may cause difficulties in driving gimbals according to the tracking result.

To verify applications for real time embedded systems, computational cost must be considered. With the computer platform Intel^®^ Core™ i5 CPU @2.27GHz, manufactured by HP (Palo Alto, CA, USA), computation costs were measured. As shown in Table 2, OA-CMT is fastest compared with other trackers for all test images. In addition, since the computation time required to perform the proposed algorithm depends on the image size, the specification of the embedded board can be determined according to the image resolution of the sensor.

## 5. Conclusions

This paper proposes an occlusion aware-coast mode tracking algorithm for infrared imagers. OA-CMT includes the decision algorithm of predicting target occlusion with image processing, the tracking filter to output predictions of target position in blind time, and post-blind time automatic re-locking for user’s convenience. The proposed algorithm helps the sensor driving unit to continue its movement during blind time caused by target occlusion.

The prediction of target obstruction is designed by checking the occlusion both of target likelihood brightness and background screening around the target considering the target’s size, moving direction and speed. For memory tracking, the α-β-γ filter is used to predict the target’s position. The target re-locking algorithm is proposed using target modeling of histogram ratio between target and background.

**Figure 23:**
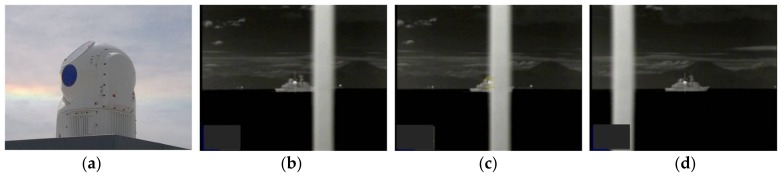
Result of application for naval EOTS: (**a**) shape of naval EOTS (**b**–**d**) operational images of testing coast mode tracking.

**Figure 24:**
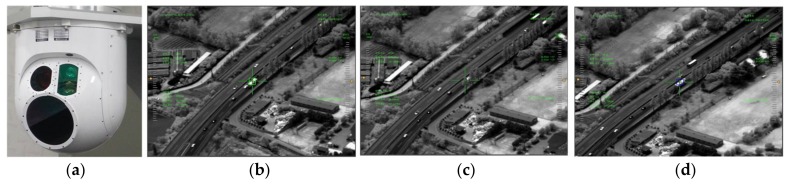
Result of application for airborne EO/IR: (**a**) shape of airborne EO/IR (**b**–**d**) operational images of testing coast mode tracking.

Computer simulations with various test imagery sequences are carried out and their results are compared to other occlusion-related published tracking algorithms, TLD, L1-APG, and SCM. Computational costs are also presented for considering real time embedded systems. After implementation on a video tracking board, the proposed algorithm is undergoing validation tests in real environment for applications for naval EOTS (shown in Figure 23) and airborne EO/IR system (shown in Figure 24) After implementation on the video tracking board of EOTS, the performance of the proposed algorithm is validated through tests under real environment.

## Figures and Tables

**Figure 1:**
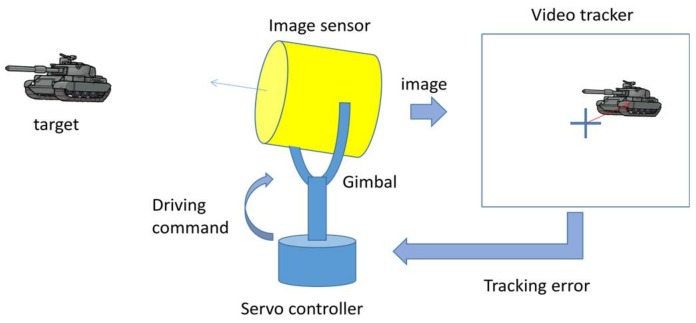
Automatic tracking procedure of an electro-optical system.

**Table 1:** Lists of the test imagery sequences.

ID	Imagery Sequences	Sensor	Obstacle Type	Description
1	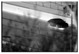	IR	Background screening	Car screened by bush
2	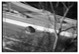	IR	Background screening	Truck screened by street lamp & trees
3	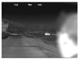	IR	Target likelihood obstacle	Tank screened by human
4	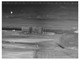	IR	Background screening	Car screened by building
5	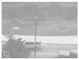	EO	Background obstacle	Car screened by trees
6	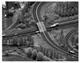	EO	Background screening	Bus screened by trees & bridge
7	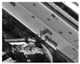	IR	Target likelihood obstacle	Car screened by traffic sign

**Table 2:** Comparison of computational cost.

ID	Image Size	Target Size	Frames Per Second			
			TLD	L1-APG	SCM	OA-CMT (Proposed)
1	720 × 480(336 frames)	56 × 48	7.05	0.03	0.98	42
2	720 × 480(831 frames)	28 × 24	6.39	0.02	1.09	30.81
3	640 × 480(130 frames)	36 × 14	3.88	2.54	2.89	71.5
4	640 × 480(283 frames)	10 × 8	10.5	3.42	1.48	71
5	320 × 240(300 frames)	90 × 40	9.87	2.92	2.18	149.5
6	1280 × 1024(310 frames)	94 × 26	2.13	2.64	1.18	6.6
7	1280 × 1024(480 frames)	24 × 16	1.37	0.3	1.43	6.67
